# *Mycoplasma phocimorsus* in Woman with Tendinous Panaritium after Cat Scratch, Denmark

**DOI:** 10.3201/eid3102.241219

**Published:** 2025-02

**Authors:** Axel Skafte-Holm, Thomas Roland Pedersen, Maria Frølund, Marc Stegger, Søren Hallstrøm, Astrid Rasmussen, Jørgen Skov Jensen

**Affiliations:** Statens Serum Institut, Copenhagen, Denmark (A. Skafte-Holm, T.R. Pedersen, M. Frølund, M. Stegger, S. Hallstrøm, A. Rasmussen, J.S. Jensen); Harry Butler Institute, Murdoch University, Perth, Western Australia, Australia (M. Stegger)

**Keywords:** Mycoplasma phocimorsus, seal finger, panaritium, doxycycline, zoonoses, bacteria, Denmark

## Abstract

A panaritium developed in a woman in Demark after her cat scratched her. Analysis of tissue by 16S rRNA gene sequencing revealed *Mycoplasma phocimorsus*, known to cause seal finger. The source of the bacterium likely transmitted by the cat is unknown, but awareness of potential zoonotic transmission from cats should be raised.

A 54-year-old woman who had a medical history of hypertension, hypercholesterolemia, and right-sided carpal tunnel syndrome sought treatment for panaritium-like symptoms in the right hand. One month before hospital admission in July 2013, the patient was scratched by her domestic cat on the right wrist and subsequently showed signs of infection. Fourteen days postscratch, the woman was treated with phenoxymethylpenicillin. Because of worsening symptoms, 1 g flucloxacillin 4 times daily was initiated on day 26 postscratch. On day 30 postscratch, the woman was hospitalized for signs of carpal tunnel infection and suspicion of underlying abscess formation. 

The patient was afebrile, and laboratory tests were marginally affected (Table). Clinicians suspected acute pyogenic tenosynovitis and performed surgical intervention for decompression, revealing severe edema and synovitis in the underlying tissue. Intravenous cefuroxime (1,500 mg) was given during surgery, and amoxicillin/clavulanic acid (500 mg/125 mg 3×/d) was initiated. The next day, a clinical assessment of the soft tissue and secondary suturing was performed, and the patient was discharged. Because of severe pain radiating toward the elbow and clawhand formation, the patient was readmitted 4 days after hospital discharge (day 35 postscratch). An acute surgical intervention involving fasciotomy of the distal forearm was performed because of suspected compartment syndrome, during which massive synovitis and serous fluid were observed. The serous fluid was collected for microbiological examination, a local gentamicin implant was applied, and intravenous cefuroxime was initiated. 

**Table:** Laboratory results detected in a case of Mycoplasma phocimorsus infection in woman with tendinous panaritium after cat scratch, Denmark*

Laboratory tests	Reference range	Day 30	Day 35	Day 38	Day 40	Day 46	Day 59
C-reactive protein, mg/L	<8.0	21	26	36	83	24	9
Leukocytes	3.50–10.00	7.10	8.9	13.8	13.0	5.30	6.10
Neutrophils	2.00–7.00	4.68	5.67	10.58	8.62	2.82	3.13
Lymphocytes	1.30–3.50	1.91	2.65	2.58	3.52	2.00	2.40
Monocytes	0.20–0.70	0.30	0.40	0.60	0.67	0.36	0.36
Eosinophils	<0.50	0.15	0.20	0.01	0.14	0.13	0.17
Basophils	<0.10	0.04	0.02	0.02	0.08	0.03	0.05

*Values are 1 × 10^9^ cells/L except as indicated.

On day 38 postscratch, antibiotic drugs were broadened to piperacillin/tazobactam (4 g/4 g 4×/d). However, laboratory results from a blood culture showed increased levels of infection markers, and the patient experienced increasing pain at day 40 postscratch, leading to surgery and application of another gentamicin implant. Tissue from the lesion was sent to Statens Serum Institut (SSI) in Copenhagen, Denmark, for additional analysis. On day 43 postscratch, the existing treatment regimen was supplemented with intravenous gentamicin (5 mg/kg), after which C-reactive protein levels decreased and the patient’s clinical condition improved by day 46 postscratch. All antibiotic drugs were discontinued on day 49 postscratch, and the patient was discharged with scheduled outpatient visits, which were unremarkable. 

In August (day 59 postscratch), analysis from SSI showed bacteria from the Mycoplasmataceae family associated with seal finger, which is a painful subacute infection typically afflicting persons’ fingers after contact with seals (*1*). Despite normal laboratory test results but increasing patient-reported pain and wound secretion, the patient was given doxycycline for 3 weeks and moxifloxacin (400 mg 1×/d) for 14 days. After completing that drug regimen, the patient continued to experience burning pain, restricted movement, and swelling of her right hand. In October, after completion of therapy, she had persistent symptoms, limiting her work as a service assistant at the local hospital to only 2 hours per day. 

After *M. phocimorsus* was identified at SSI, whole-genome metagenomics sequencing was unsuccessfully attempted from the original specimen. However, using PCR primers covering the complete 16S rRNA sequence, a near-complete sequence was obtained. At the time of diagnosis, tissue from the lesion was examined for *Mollicutes* DNA by conventional PCR using primers targeting the 16S rRNA gene (*1*) (Appendix). Analysis of the V1–V9 sequence (1,415 bp) showed the sequence from this study had the best homology (100% coverage, 99.72% identification) with *M. phocimorsus* (GenBank accession no. OQ945447), a species identified in 2023 in patients from Scandinavia who had seal finger after contact with seals (*1*) (Figure; Appendix).

**Figure:**
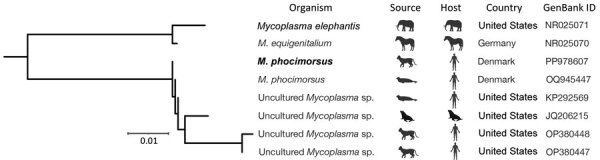
Phylogram of *Mycoplasma* species described in a case of *Mycoplasma phocimorsus* in woman with tendinous panaritium after cat scratch, Denmark. Bold text indicates species from this study. GenBank accession numbers are provided. Scale bar indicates number of nucleotide substitutions per site. ID, identification number.

In 1983, a painful swollen finger developed in a veterinary surgeon after a cat scratch. After 10 days, the infection developed into septic tenosynovitis, despite treatment with oral erythromycin. The patient was hospitalized, treated with different antibiotic drugs, and underwent multiple surgeries; however, the symptoms persisted. After 3 months, mycoplasma from the wound discharge was isolated on horse serum agar; the patient was treated with tetracycline, and symptoms ceased (*2*). A 2024 case report found 16S rRNA gene sequences of *Mycoplasma* spp. in specimens taken from a patient’s septic hand and knee after a cat bite (*3*). Alignment against the sequence reported in this study does not suggest similar species (Figure; Appendix). 

Future research should investigate whether cats are natural carriers of *M. phocimorsus* because they are carriers of *Bartonella henselae*, which is frequently found in blood and claw samples and is known to cause cat-scratch disease (*4*). Whether *M. phocimorsus* is part of the normal bacterial flora in cats or was a transient colonizer before zoonotic transmission is still uncertain. An explanation could involve wet food containing fish remnants as the source, or the cat may have been in contact with a stranded marine mammal, in which mycoplasmas are known to colonize mucosal surfaces (*5*).

In summary, this patient had prolonged hospitalization and multiple surgeries as the result of her infection. Empirically, infections are often treated with β-lactam antibiotic drugs, but they are ineffective against *Mycoplasma* spp. because these bacteria lack a cell wall (*1*). Moreover, conventional bacteriologic cultures usually yield negative results for *Mycoplasma* spp., which generally do not grow on media used for standard bacteriologic cultures because they require specialized media for growth (*1*). Consequently, healthcare providers should recognize the significance of integrating diagnostic techniques such as 16S rRNA gene analysis for bacterial identification.

Appendix Additional information on detection of *Mycoplasma phocimorsus* in woman with tendinous panaritium after cat scratch, Denmark.
