# Supplementary material for: Mycoplasma phocimorsus in Woman with Tendinous Panaritium after Cat Scratch, Denmark
Source: Emerg Infect Dis. 2025 Feb;31(2):380–2. doi: 10.3201/eid3102.241219 (PMC11845152; doi:10.3201/eid3102.241219)
Supplement: Appendix — Additional information on detection of Mycoplasma phocimorsus in woman with tendinous panaritium after cat scratch, Denmark. [file 24-1219-Techapp-s1.pdf]

Article DOI: https://doi.org/10.3201/eid3102.241219

EID cannot ensure accessibility for supplementary materials supplied by authors. Readers who have difficulty accessing supplementary content should contact the authors for assistance.

Mycoplasma phocimorsus in Woman with Tendinous Panaritium after Cat Scratch, Denmark

Appendix

Results

Tissue from the lesion was initially examined for the presence of *Mollicutes* DNA by conventional PCR by using *Mollicutes*-specific primers (GPO-3 and MGSO) targeting the 16S rRNA gene (*1*). Additional primer sets were designed for a near full-length 16S rRNA gene sequence (Appendix Table). Amplicons were gel purified (Cytiva Illustra GFX PCR DNA and Gel Band Purification Kit; Merck Life Sciences) and Sanger sequenced by Macrogen Europe B.V. Sequence assembly was performed in BioNumerics and evaluated by using BLAST. The 16S rRNA gene sequence from the specimen was identical to the gene sequence for *Mycoplasma phocimorsus* except for a single nucleotide polymorphism (C184T) and was phylogenetically distinct from other species of the genus *Mycoplasma* (Figure). Alignment showed high similarity (99% coverage and 99.64% identification) to a 16S rRNA gene sequence (1,417 bp) of an uncultured *Mycoplasma* species (GenBank accession no. KP292569) identified in an Alaskan seal hunter with septic arthritis and seal finger (*2*); thus, this sequence can be considered identical to *M. phocimorsus*. Comparison against two partial 16S rRNA sequences (315 bp), recently identified in a patient with panaritium-like symptoms (GenBank accession no. OP380448) and prosthetic joint infection in the knee (GenBank accession no. OP380447) after a cat bite, showed a sequence cover of 19% with 96.80% identity and 19% with 97.15% identity, respectively (*3*). The mycoplasma detected from this patient could not be cultivated on Friis' modified broth (*1*), Hayflick-type broth, or agar (*1*,*4*); thus, whole genome sequencing was not feasible.

References

1. Skafte-Holm A, Pedersen TR, Frølund M, Stegger M, Qvortrup K, Michaels DL, et al. *Mycoplasma phocimorsus* sp. nov., isolated from Scandinavian patients with seal finger or septic arthritis after contact with seals. Int J Syst Evol Microbiol. 2023;73:1–10. PubMed https://doi.org/10.1099/ijsem.0.006163

2. Westley BP, Horazdovsky RD, Michaels DL, Brown DR. Identification of a novel mycoplasma species in a patient with septic arthritis of the hip and seal finger. Clin Infect Dis. 2016;62:491–3. PubMed

3. Khan F, Engers D, Lieberman JA, Moudgal V. Disseminated infection with a previously undescribed mycoplasma species from a cat bite. Infect Dis Clin Pract. 2024;32:1–4. https://doi.org/10.1097/IPC.0000000000001314

4. McCabe SJ, Murray JF, Ruhnke HL, Rachlis A. Mycoplasma infection of the hand acquired from a cat. J Hand Surg Am. 1987;12:1085–8. PubMed https://doi.org/10.1016/S0363-5023(87)80119-3

Appendix Table. Primer sequences

Primers	Sequence
M.pho-54F	5′-ATACATGCATGTCGAGCGGAG
16S-806R	5′-GGACTACHVGGGTATCTAAT
M.pho-475F	5′-ATGAATTAGTCTTGACGGTACCTTGTC
M.pho-1280R	5′-TTTGAGGTTTGCTTGCCGTTA
M.pho945F	5′-AGCATGTGGTTTAATTTGAAGATACGCGTAG
M.pho-1488R	5′-GACTTCACCCCAGTCACCAGA
